# The expanded development and application of CRISPR system for sensitive nucleotide detection

**DOI:** 10.1007/s13238-020-00708-8

**Published:** 2020-04-03

**Authors:** Fengjing Jia, Xuewen Li, Chao Zhang, Xueming Tang

**Affiliations:** 1 Translational Medical Center for Stem Cell Therapy and Institute for Regenerative Medicine, Shanghai East Hospital, Shanghai Key Laboratory of Signaling and Disease Research, School of Life Sciences and Technology, Tongji University, Shanghai, 200000 China; 2 Institute of Biotechnology Research, Shanghai Academy of Agricultural Sciences, Key Laboratory of Agricultural Genetics and Breeding, Shanghai, 201106 China; 3 Crops Ecological Environment Security Inspection and Supervision Center (Shanghai), Ministry of Agriculture and Rural Affairs, Shanghai, 201106 China; 4 Silicon Gene Tech Co., Ltd., Shanghai, 200124 China

CRISPR/Cas system, originally developed as genetic editing tool, also shows great potentials for nucleotide detection. A recent study published in *Molecular Cell* (Freije et al., 2019) developed a Cas13a-based CARVER (Cas13-assisted restriction of viral expression and readout) to detect RNA viruses such as lymphocytic choriomeningitis, influenza A and vesicular stomatitis, which provided a potential expanded application for the detection of a broad range of viral nucleotides in disease diagnosis.

CRISPR/Cas (clustered regularly interspaced short palindromic repeats/CRISPR-associated) systems are utilized by bacteria and archaea as adaptive immune system to defend against phage infection. Cas effectors are guided by a CRISPR RNAs (crRNAs) to bind and cut DNA or RNA targets to defend against invading nucleotides (Horvath and Barrangou, 2010; Sorek et al., 2013; Barrangou and Marraffini, 2014). The discovery of CRISPR/Cas system dated back to 1987, the regularly spaced direct repeats were first found in the *iap* gene of *Escherichia coli* (Ishino et al., 1987). Until 2002, the spaced direct repeats were named as CRISPR (Jansen et al., 2002). In 2012, Jinek et al. reported that CRISPR/Cas9 could specifically cleave the target DNA with a single RNA chimera (Jinek et al., 2012), which opened the prelude of CRISPR/Cas9 system for genomic editing.

Since CRISPR/Cas9 was discovered, CRISPR/Cas systems attracted much attention and CRISPR toolbox had been continuously expanded. As a potent complement to DNA targeting CRISPR toolbox, CRISPR/Cas12a (previously known as CpfI), a Class 2 type V CRISPR/Cas effector, was characterized (Zetsche et al., 2015) with the capability to efficiently cleave target double-stranded DNA (dsDNA) guided by a crRNA. Moreover, differing from Cas9, Cas12a possessed a target-dependent nonspecific single-stranded DNA (ssDNA) cutting activity (Chen et al., 2018). Beyond dsDNA, ssRNA molecules could also be edited by another Cas protein, CRISPR/Cas13a (previously known as C2c2) (Abudayyeh et al., 2016). Cas13a, as a class 2 type VI CRISPR effector, was programmed to cleave the target RNA guided by crRNA. In addition, as an expanded RNA-targeting CRISPR toolbox, Cas13a owned the property for target-activated degradation of non-target RNA molecules (East-Seletsky et al., 2016). In 2018, a serial of smaller size of CRISPR/Cas14 effectors (Cas14a, Cas14b and Cas14c) were reported (Harrington et al., 2018), with the highly selective cleavage preference for ssDNA. Moreover, Cas14 possessed a target-dependent indiscriminate ssDNA cutting activity. Subsequently, CRISPR toolbox was further expanded with newly developed CRISPR/Cas systems, including Cas12b (Shmakov et al., 2015; Teng et al., 2018; Strecker et al., 2019; Teng et al., 2019), Cas12c, Cas12g, Cas12h, Cas12i (Yan et al., 2019) and Cas13d (Konermann et al., 2018; Yan et al., 2018). Recently, the emerging CRISPR tools had been developed abundantly for the application of nucleotide detection.

**Figure 1:**
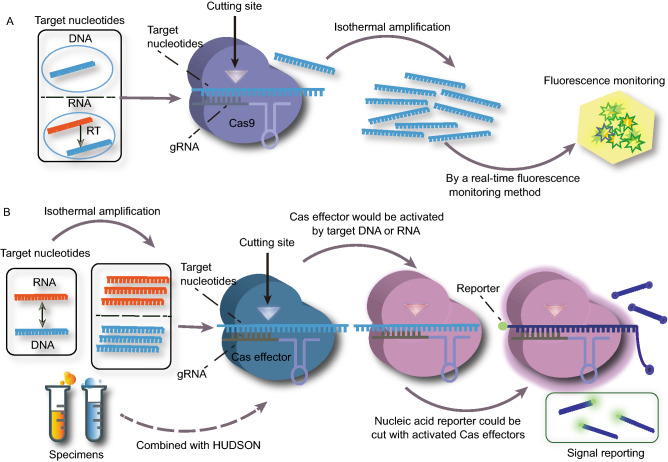
**The mechanism of CRISPR-based nucleotide detection**. (A) A class of CRISPR/Cas tools for nucleotide detection is based on specific binding and cutting activity of CRISPR/Cas9. First, guided by a guide RNA (gRNA), Cas9 proteins bind and cut DNA targets. Second, large amounts of nucleotides are synthesized by isothermal amplification. Third, the detection platforms report the presence of target nucleotides with fluorescent tracking. (B) Another class of CRISPR/Cas nucleotide detection is based on collateral cleavage of CRISPR/Cas effectors. First, a large number of nucleotides are synthesized by isothermal amplification. Second, guided by a crRNA/sgRNA, Cas effectors (Cas12, Cas13 and Cas14) recognize and cleave target nucleotides. When combined with HUDSON, the detection platforms could directly detect target nucleotides from body fluid of human patients. Third, Cas effectors are activated and cut the reporters to release visual fluorescent signal

A class of CRISPR/Cas tools for nucleotide detection is based on the specific binding and cutting activity of CRISPR/Cas9 (Fig. 1A). Pardee et al. reported a method for detection of Zika virus (Pardee et al., 2016). This study showed that the detecting capability and specificity dramatically enhanced in combination with CRISPR/Cas9. In the presence of CRISPR/Cas9, this platform could discriminate single-nucleotide resolution between various genotypes of Zika virus. In 2018, a Cas9-based nucleotide detection tool termed CAS-EXPAR (Cas9 triggered exponential amplification reaction) was developed. Combined with exponential amplification reaction, CAS-EXPAR could detect the target at concentration as low as 0.82 amol (0.82 × 10^−18^ mol/L) (Huang et al., 2018). Subsequently, this technology was optimized to monitor methylation status of DNA fragment. The changes of DNA methylation showed strong correlation with disease such as cancer (Field et al., 2018). CAS-EXPAR provided a versatile option for detection of primitive or methylated DNA molecules, an ideal method for early diagnosis of tumors. Recently, many other Cas9-based detection tools were constantly developed (Zhang et al., 2018; Zhou et al., 2018). The nuclease-deactivated mutant Cas9 (dCas9) was able to bind target DNA without cutting activity. Wang et al. developed a microRNA (miRNA) sensor consisting of dCas9, miRNA-mediated sgRNA and red fluorescent protein (Wang et al., 2019). This sensor provided an exquisite example to measure miRNA activity and track cell-state transition, so that the activity of miRNA in stem cell differentiation and cancer progression could be timely monitored (Wang et al., 2019).

Another class of CRISPR/Cas tools for nucleic acid detection is based on a target-dependent nonspecific cleaving activity (termed as collateral cleavage) of CRISPR/Cas effectors (Fig. 1B). In combination of the target-dependent indiscriminate RNA cutting activity of Cas13a with recombinase polymerase amplification, Cas13a-based SHERLOCK (specific high sensitivity enzymatic reporter UnLOCKing) was developed (Gootenberg et al., 2017) with attomolar (10^−18^ mol/L) sensitivity for detecting Zika or Dengue viruses. Strikingly, SHERLOCK, as a CRISPR based platform, had shown single-molecule sensitivity for nucleotide detection. The emergence of high sensitivity, convenience and low cost of SHERLOCK shed light on the way for nucleotide detection based on collateral effect of CRISPR/Cas system. However, a drawback of SHERLOCK made it unsuitable for quantitative detection (Gootenberg et al., 2017). Shortly afterwards, it was upgraded to SHERLOCK version 2 (SHERLOCKv2) (Gootenberg et al., 2018) with four channels to detect multiple targets in single reaction. The 3.5 times more sensitivity, as well as the quantitative, potable and visual readout made SHERLOCKv2 a powerful tool for nucleic acid detection. When paired with HUDSON (heating unextracted diagnostic samples to obliterate nucleases), SHERLOCK could directly detect Zika and Dengue viruses from bodily fluids of patients (Myhrvold et al., 2018).

Subsequently, DETECTR (DNA endonuclease-targeted CRISPR trans-reporter) with prominent property for DNA detection joined the team of nucleotide detection. DETECTR combined the target-dependent indiscriminate DNA cutting activity of Cas12a with recombinase polymerase amplification for DNA detection with attomolar sensitivity (Chen et al., 2018). It became the first tool to detect human papillomavirus (HPV) from clinically collected patient specimen. With the emergence of Cas14, the Cas14a based Cas14-DETECTR was presented (Harrington et al., 2018) and further elevated the vitality for ssDNA detection. In comparison to SHERLOCK, DETECTR became more convenient for DNA detection because SHERLOCK was originally developed for detecting target RNA and transcription from DNA to RNA must be performed beforehand.

In addition to the detection methods above, other tools had been gradually developed, such as HOLMES (one-hour low-cost multipurpose highly efficient system) (Li et al., 2018). Cas12a-based HOMLS was also capable to detect nucleic acid with attomolar sensitivity. In comparison with Cas12a, Cas12b exhibited higher activity with dsDNA. An updated version HOMLSv2, combined of Cas12b and isothermal amplification, was developed subsequently to detect nucleotide, discriminate SNP, quantify DNA and DNA methylation degree (Li et al., 2019). Unlike DETECTR, which was designed for qualitative measurement only, HOLMES could be utilized for quantitative detection. Since then, other Cas12-based detection methods had been developed and optimized (Liang et al., 2019).

Currently, RNA viruses such as Zika, Dengue, Japanese encephalitis, lymphocytic choriomeningitis, influenza A and vesicular stomatitis, and DNA viruses such as human papillomavirus and pseudorabies could be detected by SHERLOCK (Gootenberg et al., 2017), DTECTRE (Chen et al., 2018), HOLMES (Li et al., 2018) and CARVER (Freije et al., 2019). The recent emergence of Corona Virus Disease 2019 (COVID-19), caused by a novel ssRNA virus, has led to serious harm to human health worldwide. Nucleic acid detection played an important role in the diagnosis of COVID-19. Currently, molecular diagnosis of severe acute respiratory syndrome coronavirus 2 (SARS-CoV-2) had been mainly based on Real-time polymerase chain reaction (qPCR), which required expensive equipment and was time-consuming. Moreover, frequent false-negative results from qPCR seriously had delayed the treatment of patients and the prevention and control of COVID-19. In contrast, CRISPR-based tools showed better superiority for nucleotide detection because they were faster, cheaper, more sensitive and accurate within similar workload. Luckily, CRISPR-based detection methods for SARS-CoV-2 had been developed recently (Metsky et al., 2020), making it suitable for molecular diagnosis of this epidemic outbreak. Meanwhile, a rapid SHERLOCK based detection platform of SARS-CoV-2 had also been developed by Feng Zhang’s laboratory that could be completed in one hour.

In addition to detect RNA and DNA viruses from contagious diseases, CRISPR- based tools also show potentials for detection of tumor derived nucleotides such as circulating tumor DNA (ctDNA) (Jenkins et al., 2017). As a component of primary tumors into the circulatory system or other body fluids, ctDNA plays an important role in the evaluation of tumor progression and metastasis. At present, the detection of ctDNA is mainly based on PCR or NGS (next-generation sequencing). However, it is usually quite difficult to monitor subtle DNA mutations by PCR, and NGS often generates numerous false-positive results. In contrast, CRISPR-based system shows higher fidelity, sensitivity and capability to discriminate single-base mismatch, which offers a great alternative for ctDNA detection (Jia et al., 2018).

Moreover, with the global cultivation of genetically modified agricultural products and the establishment of genetically modified organism (GMO) labeling management system, the detection of genetically modified components is becoming more and more important in the agricultural field (Kebed, 2015). The advantages of CRISPR tools including high sensitivity, convenience and low cost show great potentials to replace existing outdated techniques for GMO detection. In combination of Cas9 with rolling circle amplification and gold nanoparticles, CRISPR-based method for plant pathogens could visually detect target DNA at concentration as low as 2 pmol/L (10^−12^ mol/L), which could provide a convenient platform for crops inspection (Chang et al., 2019). Furthermore, a modified version of SHERLOCK, designed to detect multiple soybean genes in one single reaction has been recently developed to monitor the traits of crops during breeding (Abudayyeh et al., 2019). African swine fever (ASF), caused by infection of African swine fever virus (ASFV), becomes an infectious disease with high fatality rate in most pig farms. The point-of-care detection by farmers is of great significance for the prevention and diagnosis of ASF. At present, the detection of ASFV is mainly based on qPCR, which is time-consuming and requires equipping expensive instruments. To improve this situation, Cas12 and Cas14-based detection method for ASFV has been developed to target three key genes of ASFV (VP72, K205 R, CP530R). It could visually detect ASFV from pig secretions such as blood, urine and nasal swab within 15 min (unpublished data). We can envision the prospects of broad applications of CRISPR-based detecting systems in the agriculture, especially the on-site evaluation of transgenosis of genetically modified foods and more infectious animal diseases in the near future.

CRISPR-based tools are now in a state of full bloom. In comparison with Cas9, other Cas effectors possessing collateral activity are becoming most popular due to the innate technical advantages. Cas12 effectors exhibit more expertise in detecting tumor associated viral markers, such as HPV (Chen et al., 2018). Cas13 effectors show the talent on RNA viral detection, such as Zika and Dengue (Gootenberg et al., 2017). Cas14 effectors show superiority in ssDNA detection (Harrington et al., 2018). Different Cas platforms could be selected and utilized for distinct areas based on their individual talents (Table 1).

**Table 1:** The characteristics of representative CRISPR-based detection platforms

Name	Version	CRISPR system	Target	Refs	The performance of detection			
					Attomolar sensitivity	Quantitative measurement	MMultiplexed detection	Visualized readout
CAS-EXPAR		Cas9	DNA/RNA	Huang et al., (2018)	**√**			
SHERLOCK	v1	Cas13a	DNA/RNA	Gootenberg et al., (2017)	**√**			
	v2	Cas13a, Cas13b, Cas12a	DNA/RNA	Gootenberg et al., (2018)	**√**	**√**	**√**	**√**
DETECTR	vCas12	Cas12a	DNA	Chen et al., (2018)	**√**			
	vCas14	Cas14a	DNA	Harrington et al., (2018)				
HOLMES	v1	Cas12a	DNA/RNA	Li et al., (2018)	**√**			
	v2	Cas12b	DNA/RNA	Li et al., (2019)	**√**	**√**		

The various CRISPR-based platforms provide inspirations on how to detect nucleotide efficiently, sensitively and conveniently. For example, by combining with multiple members of Cas effectors, the detection tools possessed multiple channels to detect several targets in a single reaction. This feature makes it expandable for high-throughput screening system. By combining with Csm6, a CRISPR type III protein, the sensitivity of signal detection could be further elevated (Gootenberg et al., 2018). The detection platforms with additional lateral flow for visual readout show significant improvement for the control of the spread of infectious diseases, especially in certain areas without essential instruments for molecular diagnostics. By adding amplified enzymes to the detection system, the nucleotide amplification and detection steps have been integrated into a reaction system. This procedural improvement during sample preparation could effectively avoid cross-contamination.

Several constraints for CRISPR-based detection tools still remain to be resolved. First, the off-target effects of CRISPR may affect the accuracy for nucleotide detection. Recently, the method of prime editing has been developed by combining impaired Cas9 protein with reverse transcriptase,by which specific DNA targets could be synthesized (Anzalone et al., 2019). Prime editing technique not only shows significance for genetic editing, but also provides an ingenious path to enhance the efficiency of CRISPR-based detection methods. Second, CRISPR/Cas effectors exhibit varying degrees of tolerance to mismatches between the guide RNA and the target nucleotides, which may also affect the accuracy of detection. Therefore, optimized technique needs to be invented to solve these issues. In short, higher accuracy, sensitivity, convenience and lower cost should be the future focus and breakthrough for CRISPR detecting systems.

As a summary, these emerging CRISPR/Cas detection tools show great potentials in the detection of viral and tumor derived nucleotides, DNA methylation, and single-nucleotide polymorphism. Because of its advantages, CRISPR-based methods would be more suitable for molecular diagnosis of major epidemic outbreaks than conventional techniques. Moreover, CRISPR-based tools could also be expanded for detection in many other fields such as agriculture and aquaculture. With the continuous expansion of CRISPR toolbox, and the rapid development of CRISPR-based platforms, more and more fields will definitely benefit from CRISPR/Cas utilization in the future.
